# Supplementary material for: Complete genomes of a multi-generational pedigree to expand studies of genetic and epigenetic inheritance
Source: bioRxiv. 2025 Dec 16:2025.12.14.693655. Preprint. [Version 1] doi: 10.64898/2025.12.14.693655 (PMC12746033; doi:10.64898/2025.12.14.693655)
Supplement: Supplement 3 [file media-3.zip › washu-pedigree supplementary files/Cytogenomics Report/HG06803B P4.pdf]

CORIELL INSTITUTE FOR MEDICAL RESEARCH

Cytogenomics Chromosome Analysis Report

**Coriell Case ID:**	**HG06803*B**
**Cell Line ID:**	**MGISTL-PAN010**
**Passage:**	**4**
**Specimen Type:**	**Lymph**
**Species:**	**Human**
**Date Received:**	**08/24/2021**
**Banding Technique:**	**G-banding**
**Cells Counted:**	**20**
**Cells Analyzed:**	**5**
**Cells Karyotyped:**	**5**

ISCN: 46,XX[19]

Additional Information: One additional cell was 46,XX,del(20)(q12)[1].

Small chromosome anomalies and mosaicism may not be detectable using the standard methods employed. Chromosome analysis was performed at a level of 400 bands or greater.

This analysis was performed for research purpose only.

Reviewed by:
Access Genomics, LLC by Yoshiko Mito, PhD, FACMG Date 9/12/2021
Cytogenomics Consultant for Coriell Institute for Medical Research

403 Haddon Avenue, Camden, NJ 08103-1505 | (856) 966-7377 TEL | (856) 964-0254 FAX | cytogenetics@coriell.org

CORIELL INSTITUTE
FOR MEDICAL RESEARCH

Cell Line ID: HG06803*B

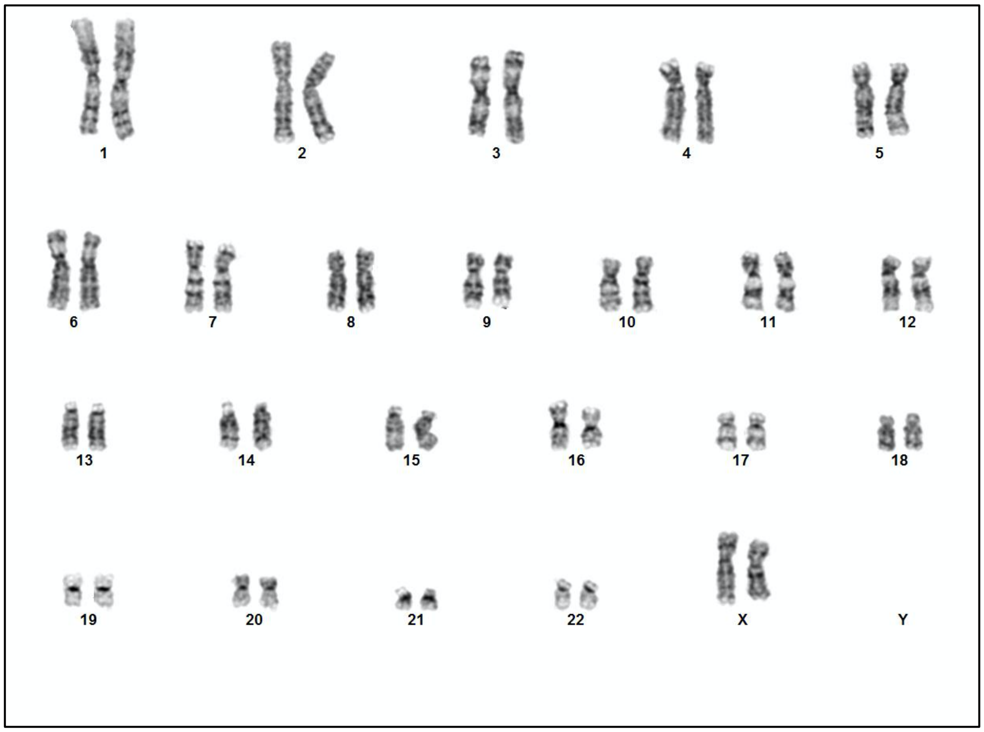

46,XX

Cell Line ID: HG06803*B

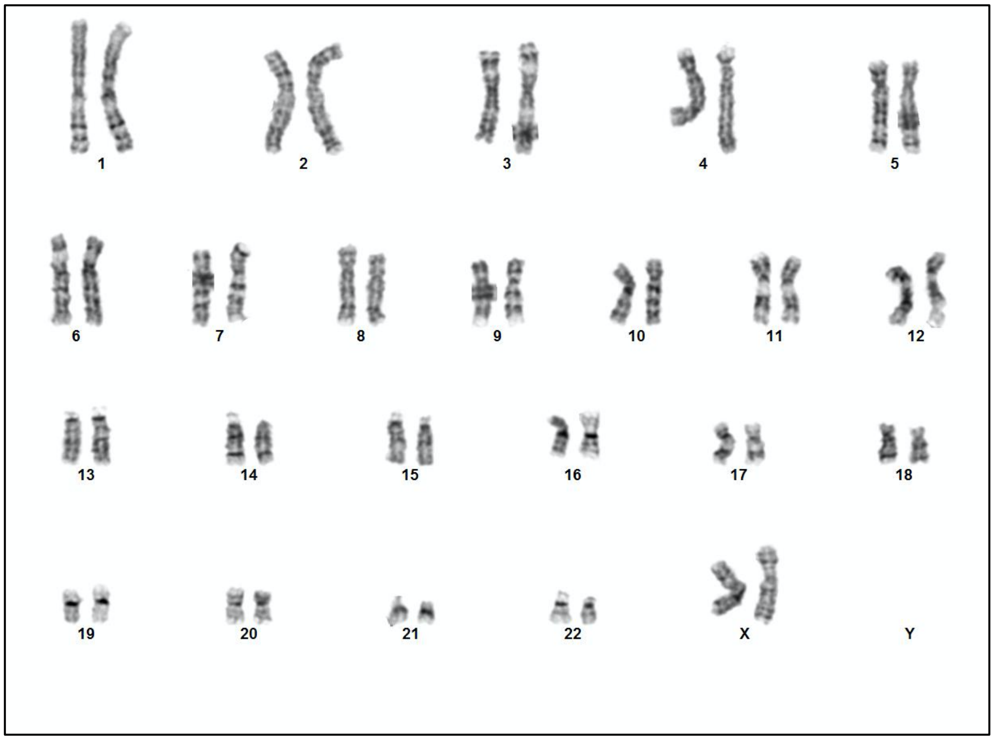

46,XX

403 Haddon Avenue, Camden, NJ 08103-1505 | (856) 966-7377 TEL | (856) 964-0254 FAX | cytogenetics@coriell.org
